# More Than Meets the Eye Regarding Cancer Metabolism

**DOI:** 10.3390/ijms22179507

**Published:** 2021-09-01

**Authors:** Anna Kubicka, Karolina Matczak, Magdalena Łabieniec-Watała

**Affiliations:** 1 Department of Medical Biophysics, Faculty of Biology and Environmental Protection, Institute of Biophysics, University of Lodz, Pomorska Street 141/143, 90-236 Lodz, Poland; magdalena.labieniec@biol.uni.lodz.pl; 2 Doctoral School of Exact and Natural Sciences, University of Lodz, Banacha Street 12/16, 90-237 Lodz, Poland

**Keywords:** Warburg effect, cancer metabolism, tumour heterogeneity, glycolysis, glutamine, lactate

## Abstract

In spite of the continuous improvement in our knowledge of the nature of cancer, the causes of its formation and the development of new treatment methods, our knowledge is still incomplete. A key issue is the difference in metabolism between normal and cancer cells. The features that distinguish cancer cells from normal cells are the increased proliferation and abnormal differentiation and maturation of these cells, which are due to regulatory changes in the emerging tumour. Normal cells use oxidative phosphorylation (OXPHOS) in the mitochondrion as a major source of energy during division. During OXPHOS, there are 36 ATP molecules produced from one molecule of glucose, in contrast to glycolysis which provides an ATP supply of only two molecules. Although aerobic glucose metabolism is more efficient, metabolism based on intensive glycolysis provides intermediate metabolites necessary for the synthesis of nucleic acids, proteins and lipids, which are in constant high demand due to the intense cell division in cancer. This is the main reason why the cancer cell does not “give up” on glycolysis despite the high demand for energy in the form of ATP. One of the evolving trends in the development of anti-cancer therapies is to exploit differences in the metabolism of normal cells and cancer cells. Currently constructed therapies, based on cell metabolism, focus on the attempt to reprogram the metabolic pathways of the cell in such a manner that it becomes possible to stop unrestrained proliferation.

## 1. Introduction

Features that distinguish tumour cells from normal cells are increased proliferation, impaired differentiation and maturation, which result from regulatory changes occurring at cellular and tissue levels in the tumour. Impaired access to nutrients such as glucose and oxygen, among others, manifests itself in impaired cellular metabolism, resulting in a switch to anaerobic metabolism. Warburg in 1927 studied cancer cells for respiration and fermentation processes. He observed that, in the presence of oxygen, glucose is converted to pyruvate and then enters the tricarboxylic acid cycle (TCA cycle), where it should undergo oxidative phosphorylation, and, consequently, lactate production should be minimal. However, Warburg, in his in vivo and ex vivo studies, showed that the presence of oxygen increased glucose uptake and increased lactate synthesis in cancer cells compared to normal cells [1]. This process, called the “Warburg effect”, demonstrates that, compared to normal cells, cancer cells prefer glycolysis to mitochondrial oxidative phosphorylation to generate the energy required for growth. This process is commonly found in proliferating cells and occurs even when the tissue is adequately oxygenated, whether normal or cancerous. However, the most important task for rapidly growing tissues is to produce components such as nucleotides, amino acids and lipids, which are essential for the production of stem cells, and not necessary to produce large amounts of ATP. In biomass production, on the other hand, the main substrate is pyruvate, which has its origin in glycolysis and is further converted to acetyl-coenzyme A [2]. Meanwhile, the “Warburg effect” alone does not fully explain how aerobic glycolysis causes mass accumulation and excessive proliferation of cancer cells. This is because glucose does not necessarily supply all the elements needed for cell growth. When it comes to cellular components, other elements such as nitrogen, sulphur and phosphorus are obviously important, in addition to carbon, oxygen and hydrogen [3]. A developing tumour can be seen as a structure consisting of many cells that do not have enough nutrients and oxygen. Because of this, new blood vessels can form and grow. With the formation of new blood vessels inside the tumour, better oxygenation and nutrition of the cells is possible, which promotes metastasis [4]. There is still no good explanation why mitochondrial respiration is abandoned in the cancer cell in favour of aerobic glycolysis. Mitochondrial oxidative phosphorylation from one glucose molecule produces 32 to 36 ATP molecules, whereas glycolysis produces only 2 ATP molecules. One hypothesis is that lactate production in cancer cells is associated with impaired oxidative phosphorylation due to mitochondrial damage [5]. A polemic heated up around Warburg’s theory, particularly involving Sidney Weinhouse. Using isotope labelling, Weinhouse showed that oxidative phosphorylation rates in normal cells, as well as in cancer cells, are similar, indicating that mitochondria in cancer cells are not damaged [6]. Nevertheless, the production of ATP by glycolysis is more efficient because this route is faster than oxidative phosphorylation, if the glucose level is unlimited.

## 2. Warburg Effect

Normal cells undergoing division use oxidative phosphorylation as their main energy source. As mentioned before, one glucose molecule can then become a source of as many as 32 to 36 ATP molecules, whereas in the case of metabolism based solely on glycolysis, the gain is only 2 ATP molecules per one glucose molecule [7]. Although the aerobic metabolism of glucose is more efficient, the metabolism based on intensive glycolysis provides intermediate metabolites, necessary for the synthesis of nucleic acids, proteins and lipids, for which there is a continuous high demand due to intensive cell division [8]. Glucose metabolism in the pentose phosphate pathway (PPP) is accompanied by the production of NADPH, which is not only necessary for reduction biosynthesis but also for maintaining optimal levels of reduced glutathione (GSH), which plays a major role in protecting the cell against reactive oxygen species (ROS) [9]. The concentration of ROS in cancer cells is usually high, which promotes damage to DNA and tumour progression, with their toxic effects on all cell structures and proapoptotic effects, making it necessary to keep ROS in the tumour cell constant [10]. Furthermore, reactive oxygen species can induce apoptosis in glucose-free cells, as their high concentration inhibits the oxidation of fatty acids, an alternative energy source in the absence of sugar [11]. Another important metabolic factor for the progression of cancer is acidification, occurring due to the excessive production of lactate—the final product of anaerobic glycolysis. The lactate is secreted into the extracellular space, causing local acidosis [12]. Low pH in the microenvironment of the tumour causes death of the surrounding normal cells, degradation of the intercellular matrix and induction of angiogenesis, which increases the aggressiveness of the tumour. The determination of lactate dehydrogenase (LDH) concentration is a well-known prognostic marker for many cancer types [13].

## 3. Crabtree Effect

The switch from aerobic metabolism to glycolysis is observed in many cancers and is induced by glucose. This apparent phenomenon is known as the Crabtree effect, and has not yet been well understood [14]. The basic hypothesis states that ADP is utilized by the glycolysis enzyme competitively with mitochondria. Thus, when ADP is an essential substrate for oxidative phosphorylation, it may lead to the inhibition of ATP synthase as a result of intensive glycolysis in mitochondria, thereby inhibiting the respiratory chain reaction. Nevertheless, it is likely not a leading factor for the Crabtree effect, given that the Km value for adenine nucleotide translocase (ANT) is about 100 times lower than those for phosphoglycerate and pyruvate kinase [15]. This may indicate that with intensive glycolysis, mitochondria may still use ADP, which is transported from cytosol. Nevertheless, experimental studies have shown that the inorganic phosphate supply eliminates the Crabtree effect. This is of course confirmed by the fact that when glucose was added, there was a marked reduction in Pi levels in the tumour cells. Consequently, it can be concluded that the thermodynamic potential of phosphate [ATP/ADP/Pi] may actually be relevant for its induction [16]. There is another hypothesis that fructose-1,6-bisphosphate plays an important role in the Crabtree effect [17].

## 4. Metabolism in Normal and Cancer Cells

Cellular respiration is a catabolic process and involves the conversion of complex organic compounds into simpler ones, with the production of energy in the form of ATP. The energy obtained this way is used by organisms for vital functions such as maintaining a constant body temperature, growth and movement. The substrate that plays a key role in cellular respiration is glucose. In aerobic respiration, pyruvate enters the mitochondrion where it is further metabolised in the presence of oxygen [18].

**Figure 1:**
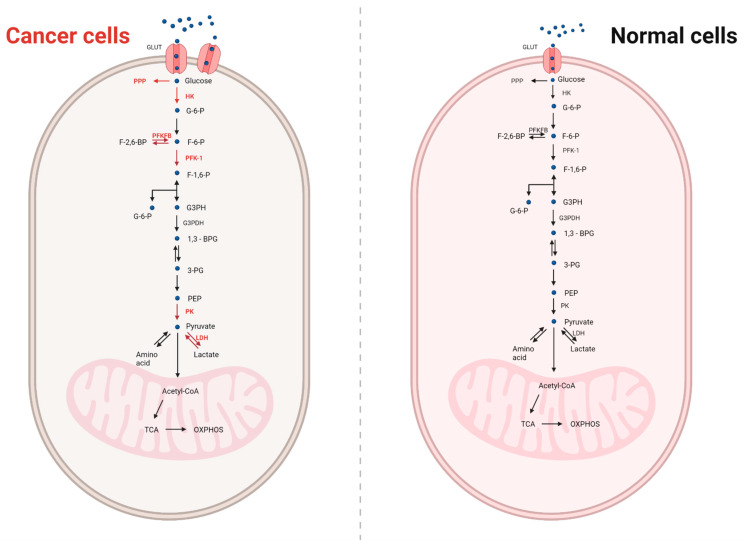
Comparison of cancer and normal cell metabolism. The glucose molecule is transferred to the cells by glucose transporters (GLUT). In normal cells, glucose is metabolised to two pyruvate molecules. Pyruvate in the presence of oxygen passes to the mitochondrion, where it is oxidized to acetyl-CoA and then incorporated into the Krebs cycle. Nevertheless, the pentose phosphate cycle can be an alternative glucose metabolism. The main function of this process is the production of NADPH. In cancer cells, the expression of glucose transporters is increased, and anaerobic respiration is encouraged, even with optimal oxygen availability. A significantly increased activity of some glycolytic enzymes and phosphofructokinase 2 and an increased pentose phosphate cycle activity are observed. The increase in activity is marked in red. Created with BioRender.com.

On the other hand, in the case of anaerobic respiration, organisms obtain energy from the transformation of organic or inorganic compounds without oxygen. Both types of respiration have in common the initial transformation known as glycolysis, which occurs without the presence of oxygen in the cytoplasm of the cell. During glycolysis, however, a glucose molecule is converted by the enzyme pyruvate kinase into two pyruvic acid molecules. In turn, the fermentation process that takes place in the cytoplasm of the cell is variously named, depending on the product that is produced in this process. The most commonly mentioned are alcoholic and lactic fermentation. Lactic acid is formed, among others, in muscles during increased physical effort, whenever the demand for energy coincides with a temporary lack of oxygen. Following these conditions, an acetate is formed which combines with coenzyme A to form acetyl-CoA. In the reaction cycle, CO_2_ is formed from acetyl-CoA, and hydrogen is transferred to NAD and FAD to later produce ATP molecules by reaction with oxygen (Figure 1) [19].

## 5. The Metabolic Heterogeneity of Tumours

The main metabolic feature of cancer cells was for a long time considered to be the Warburg effect, according to which cancer cells in the presence of oxygen undergo glycolysis to produce lactic acid—a process referred to as aerobic glycolysis. While this remains valid, it has been known for some time that the metabolic phenotypes of cancer cells are characterised by much greater variability and diversity [2]. Indeed, the genetic instability of cancer cells is responsible for the appearance of numerous metabolic phenotypes. Metabolic diversity, which is the source of many phenotypes, provides cancer cells with an extremely valuable advantage in the context of the fight for life. This in turn explains how complex it is to develop effective therapies for many cancers. Key features at the heart of establishing metabolic phenotypes are the hostile tumour environment, hypoxia, low pH and low nutrient concentrations. Research suggests that through alternative metabolic pathways, cancer cells can adapt to a diverse supply of nutrients and oxygen, to provide energy and substances necessary for survival. Some of these metabolic pathways include fatty acid oxidation, lipid scavenging, alternative pathways and cellular respiration processes in cancer cells under hypoxia and normoxia [20,21]. Due to the lack of nutrients and oxygen, the internal conditions of the tumour microenvironment (TME) promote a metabolic change favourable to tumours that helps them to survive under these harsh conditions. During hypoxia, oxidative phosphorylation and other oxidative reactions are reduced. This condition disrupts the maintenance of redox balance and negatively affects cell signalling. Furthermore, raised reactive oxygen species (ROS) levels can cause lipid, protein and DNA damage, a condition known as oxidative stress. Due to reduced oxygen partial pressure, energy production in hypoxic cells depends on anaerobic glycolysis, while reduced oxygen levels ensure ATP production by oxidative phosphorylation [22,23].

Darwin’s theory of evolution provides a sound basis for investigation to understand how the differentiation of metabolic phenotypes occurs in cancer cells. Tumours have distinct genetic and metabolic phenotypes due to different environmental factors, e.g., vascularisation, oxygen supply and other factors. Some subgroups with particular metabolic phenotypes may prove susceptible to the use of a particular inhibitor, while other subgroups may be resistant to the drug. Hence, patients may not respond to treatment after a successful series of treatments during which most of the tumour has been eradicated but small portions have not. This selectivity contributes to the growth and development of genetically, as well as epigenetically, diverse strains, which, in turn, results in the development of different metabolic phenotypes within them [5,24].

Another theory of cancer cell heterogeneity that is worth considering is that of cancer stem cells (CSCs). In contrast to the theory of clonal evolution (where states that differ genetically and metabolically arise from a previously developed population of cancer cells as a result of population expansion, genetic diversification and the selection of some subclones over others [25,26]), the theory of cancer stem cells (CSCs) posits that CSCs which are undifferentiated and have a high rate of division are an important source of cancer cell heterogeneity. The metabolic phenotypes of the aforementioned cancer stem cells are usually diverse due to the fact that they transform into various cell types [27,28]. Notably, inside a tumour, CSCs can divide into metabolically and functionally heterogeneous subclones while remaining resistant to treatment. This is supported by findings suggesting that more differentiated cancer stem cells tend to have a better prognosis due to their lower tumorigenic potential [29]. It follows that some therapies used in cancer patients induce differentiation of CSCs. CSCs can arise both from cancer cells that have acquired stem cell properties and from differentiating stem cells that have simply accumulated mutations that transform them into CSCs [30].

## 6. The Metabolic Heterogeneity of Cancer Is Due to the Difficult Conditions

### 6.1. Effect of Hypoxia on Metabolism

A characteristic feature of 50–60% of solid tumours is a change in oxygen concentration [31,32]. This is caused by rapid cell proliferation with a concomitant deficiency in the production of newly formed vessels supplying the tumour tissue [33]. The occurrence of hypoxia is associated with poor prognosis, as well as resistance to cancer treatment. Oxygen deficiency causes increased activation of the factor hypoxia-inducible transcription factor-1 (HIF-1). The alpha subunit of this protein is not degraded in proteasomes and the expression of this factor increases [34,35]. HIF-1 mediates the induction of the expression of proteins, including the glucose transporters GLUT1 and 3, glycolytic enzymes, isoforms of lactate dehydrogenase (LDHA) and the lactate transporter monocarboxylate transporter 4 (MCT4) with properties that adapt cellular metabolism to reduced oxygen [36,37]. HIF-1 plays a very important role in hypoxia by reducing the production of acetyl-CoA, which is formed during glycolysis. This process occurs through the induction of pyruvate dehydrogenase kinase (PDK1), which, by inhibiting pyruvate dehydrogenase (PDH), causes an increase in the amount of pyruvate supplied for lactate synthesis [38,39,40]. The effect of this may be the ability of cells to switch to reductive glutamate carboxylation to generate the acetyl-CoA necessary for fatty acid synthesis. A study was conducted using 13C-carbon and showed that in normoxia, cells produced most of the acetyl-CoA using carbon from glucose, but did not use glutamine for this purpose [41,42,43]. Under hypoxia, on the other hand, the switch from glucose to acetyl-CoA is reduced, with a concomitant increase in the glutamine fraction of acetyl-CoA. Interestingly, it appears that acetate may provide one additional source of acetyl-CoA. Acetyl-CoA is produced from acetate by the cytoplasmic acetyl-CoA synthetase (ACSS2). Under hypoxia, ACSS2 expression is induced, leading to an increase in the contribution of acetate as a source to lipid biomass production. Furthermore, ACSS2 promotes HIF-2 acetylation, thereby increasing HIF-2 transcriptional activity, which, in turn, contributes to increased motility and invasion of cancer cells [44]. Under hypoxia, there is an increased production of ROS in complexes I and III of the respiratory chain due to a reduced supply of oxygen, which is a substrate for complex IV [45,46]. HIF-1 adapts the respiratory chain activity to hypoxia by modifying the expression of cytochrome c oxidase (COX). As a result of HIF-1 action, the expression of the COX4-2 isoform, which has an increased affinity for oxygen, is induced, with concomitant proteolytic degradation of the COX4-1 isoform [47]. The effect of HIF-1 is to preserve mitochondrial ATP synthesis while reducing respiratory chain ROS production. Another mechanism of reduction of mitochondrial ROS synthesis by HIF-1 is inhibition of the expression of the transcription factor c-Myc [48]. Hypoxia promotes the formation of ROS in the respiratory chain, although it is the reduced oxygen availability that is responsible for the resistance of tumours to radiotherapy [49]. Studies have shown that cancer cells undergoing hypoxia show increased resistance to radiotherapy treatment due to the fact that reduced oxygen availability results in less ROS formation during exposure to ionising radiation [50]. Another important source of energy is lactate synthesised by cells, which can exhibit antioxidant effects. Another important role of HIF-1 is to restore an adequate supply of oxygen, nutrients and building blocks to cells. This is carried out through the induction of transcription of proteins involved in angiogenesis: vascular endothelial growth factor (VEGF), inducible isoform of nitric oxide synthase (iNOS) and haem oxygenase 1 (HO-1) [51,52].

Interestingly, oxygen deficit is not necessarily a determinant of reduced cancer cell proliferation. During hypoxia, the concentration of building and energy compounds in the tumour environment decreases [53]. Thus, it can be concluded that low oxygen concentration is a signal for the adaptation of cellular metabolism to the simultaneous deficiency of energy and building blocks compounds. The alternative activity of the glutaminolysis process ensures that citrate can be synthesised, bypassing the dehydrogenases of the Krebs cycle and, therefore, without the involvement of the respiratory chain, which is the source of many ROS in hypoxia. This is a mechanism that compensates for the reduced production of acetyl-CoA from glycolysis-derived citrate. The production of lactate during hypoxia as a result of glutaminolysis is reduced due to the lower amount of L-Gln that is present in the tumour environment and, in addition, due to the downregulation of c-Myc activity by the factor HIF-1, which results in the inhibition of glutaminolysis, with a concomitant reduction in the number of mitochondria. This mechanism adjusts tumour cell metabolism to the reduced availability of oxygen, nutrients and building blocks and protects against oxidative stress resulting from the increased production of ROS in the respiratory chain. In hypoxia, cellular metabolism is switched into a standby mode so that aerobic conditions are restored and glycolysis becomes the main survival pathway [54,55,56,57]. According to studies, during hypoxia, there are two alternative glutaminolysis pathways located in the cytoplasm and mitochondrion. Both types of glutaminolysis are based on the reductive carboxylation of α-ketoglutarate to isocitrate, which is then converted to citrate. However, both reactions are directed in the opposite direction to the Krebs cycle: isocitrate dehydrogenase (IDH2) and cis-aconitase (ACO2) are involved in the mitochondrion, cytoplasmic isoforms of these enzymes IDH1 and ACO1 are involved in the cytoplasm [41,58].

### 6.2. Normoxia’s Impact on Metabolism

During normoxia, HIF-1α is rapidly degraded, with the involvement of ubiquitin and with activation of the tumour suppressor protein pVHL [59]. Despite the lack of HIF-1α activity during normoxia in cancer cells, glycolysis is a very active pathway [60,61]. Interestingly, in normal cells, MYC expression is tightly controlled. Unfortunately, increased expression of the c-Myc gene and protein occurs in approximately 70% of human cancers, including most cancers that are highly prevalent, such as breast, colorectal and prostate cancer [48,62,63,64].

The c-Myc protein is a transcription factor involved in processes related to cell growth and proliferation. The c-Myc factor directly activates genes of glycolytic enzymes such as hexokinase 2, phosphofructokinase-1 and enolase-1, and the GLUT1 transporter. Furthermore, c-Myc is an activator of LDHA expression in normoxia, resulting in the maintenance of lactate synthesis despite a reduced supply of pyruvate, which, under these conditions, is a substrate of pyruvate dehydrogenase not inhibited by PDK1 [65,66]. Thus, Myc is able to stimulate genes that increase glucose transport and metabolism. It is worth noting that, under the influence of ENO1, there is an alternative translation initiation product MBP-1, which constitutes a negative regulator of c-Myc expression. Thus, a negative feedback loop is formed and regulated by hypoxia [67]. In view of the fact that glycolytic genes show sensitivity to hypoxia-inducible factor (HIF-1) [68], there is an interaction between Myc and HIF through genes regulated by both transcription factors [69]. These studies show that HIF-1 co-activates with Myc genes related to glucose transporter and glycolysis. Notably, under conditions of hypoxia, the mentioned genes are activated by HIF-1, whereas Myc regulates the same set of genes under normoxic conditions. These observations suggest that Myc may play an important role in the Warburg effect [70].

**Figure 2:**
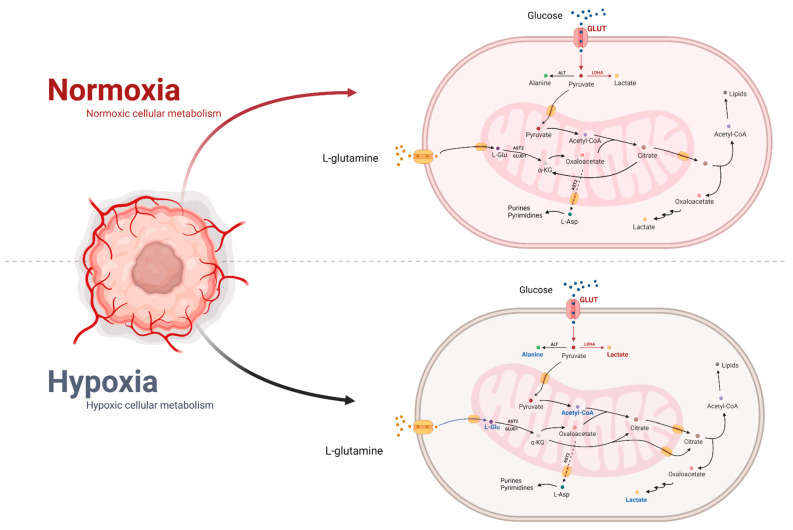
The impact of hypoxia and normoxia on glucose and glutamine metabolism in cancer cells. The increase in activity is shown in red and the decrease in blue. Adapted from “Cancer Metabolism”, by BioRender.com (2021). Retrieved from https://app.biorender.com/biorender-templates.

For rapidly dividing cells, which include cancer cells, glutaminolysis plays an important role in L-glutamine (L-Gln) metabolism during normoxia. It provides not only energy but also substrates for the synthesis of nucleic acids, proteins and lipids [49,71]. Glutaminolysis takes place in the mitochondria with the participation of Krebs cycle enzymes. In the first reaction, L-Gln is converted to L-Glu by the enzyme glutaminase with increased activity in numerous cancer types. Glutamate dehydrogenase 1 (GLUD1) and aspartate aminotransferase 2 (AST2) convert L-Glu to α-ketoglutarate, which then undergoes oxidative decarboxylation in the Krebs cycle [72]. The role of AST2 in this pathway is dual, as it synthesises both the intermediate compound α-ketoglutarate and the glutaminolysis product L-Asp, which after transfer to the cytoplasm is used for the synthesis of purines and pyrimidines [73]. In normoxia, citrate synthesis is also efficient due to the delivery of acetyl-CoA by active pyruvate dehydrogenase. Once translocated to the cytoplasm, citrate becomes a substrate for ATP citrate lyase, which reconstitutes acetyl-CoA and oxaloacetate. The resulting oxalic acid becomes a substrate for the synthesis of lactate. The role of the previously mentioned enzymes is to reconstitute NAD+ in the cytoplasm, allowing efficient glycolysis and the formation of NADPH necessary for lipid and DNA synthesis and for the cell’s antioxidant systems [74]. c-Myc activates glutaminolysis by increasing the expression of glutamine transporter genes and the glutaminase gene. This allows the initiation of the pathway by bringing glutamine into the mitochondria and deamidating it to glutamate. c-Myc also increases the number of mitochondria that contain most of the glutaminolysis enzymes (Figure 2) [49].

## 7. Effects of Oncogenes on Metabolism

Despite the high heterogeneity of cancer, metabolic reprogramming appears to involve a set of pathways that promote anabolism, catabolism or redox homeostasis. In addition to the fact that increased glucose uptake, overexpression of fatty acid synthase, more efficient aerobic glycolysis or glutaminolysis contribute to the intrinsic and/or acquired resistance to chemotherapy, metabolic reprogramming in response to conventional chemotherapy has also been described [75]. Studies have indicated that oncogenes are important factors responsible for metabolic reprogramming and, thus, the acquisition of resistance by cancer cells [76]. Although the role of oncogenes and their influence on metabolic pathways are only beginning to be appreciated in the context of metabolic modification and cancer control, the discovery that they can promote aerobic glycolysis and activate a number of pathways has contributed to the initiation of clinical trials against cancer (Table 1).

Oncogenic KRAS (Kirsten rat sarcoma viral oncogene homolog) is present in approximately 30% of all human cancers [86]. Gene expression analysis has shown that it increases the expression of, among others, the glucose transporter GLUT1, and induces the expression of hexokinase 1 and 2, phosphofructokinase-1, enolase 1 and LDHA to increase glycolytic activity [87,88,89]. Furthermore, KRAS can affect the gene expression of enzymes involved in glutaminolysis, for example, by affecting glutamate oxaloacetate transaminase 1 and 2 (GOT1 and GOT2); thus, KRAS facilitates the production of aspartate for nucleotide biosynthesis and enables the production of NADPH. Interestingly, RAS (Rat sarcoma virus)-stimulated cancer cells under metabolic stress conditions promote the uptake of lysophospholipids, which are then used to produce ATP [90,91].

One of the most intensively studied signalling pathways in cancer cells is the PI3K (phosphoinositide 3-kinase) pathway. The PI3K pathway, which is one of the key regulators of phosphoinositide metabolism, is a potential target for preclinical and clinical research conducted to reduce cancer development [92]. The most common factors contributing to the activation of this pathway are:

- Mutations in PI3K component genes;
- Mutations in tumour suppressor genes;
- Signalling by receptor tyrosine kinase [93].

According to studies, an important role in the activation of glycolysis is played by the PI3K signalling pathway. This, in turn, means that activation of the PI3K pathway allows cells to become dependent on high glucose levels. The activation of PI3K is followed by the activation of Akt, which, in turn, promotes glycolysis by enhancing the protein expression involved in this process: ectonucleoside triphosphate diphosphohydrolase 5, pyruvate kinase M2, hexokinase 2 and phosphofructokinase [94,95]. In addition, Akt is responsible for activating FOXO3a (Forkhead box protein O3), resulting in the inhibition of apoptosis and increased mitochondrial biogenesis to sustain cell growth [19]. Furthermore, Akt can also activate mammalian target of rapamycin (mTOR)—stimulating HIF-1α synthesis in normoxic states, which further increases the expression of such glycolytic enzymes as GLUT1, lactate dehydrogenase B (LDHB) and pyruvate kinase M2 (PKM2) [95]. In addition, mTOR activation further stimulates protein and lipid biosynthesis, ensuring that cells have a continuous supply of intracellular energy and nutrients. The PI3K/Akt signalling pathway, which is often mutated and overactive in cancer cells, remains crucial for the correct functioning of cells. The effects of the above-mentioned Akt activation include effects on cell size, increased glycolysis activity and enhanced cell survival [96,97].

The TP53 gene encodes a tumour suppressor protein responsible for cell cycle arrest, apoptosis or cellular ageing. It is one of the most frequently mutated genes found in many cancers [98]. However, findings also indicate that TP53 plays an important role in regulating energy metabolism and antioxidant defence. The target gene that mediates the suppressive properties of tumour protein 53 (p53) is glutaminase 2 (GLS2). GLS2 increases intracellular levels of glutamate and α-KG, which leads to increased mitochondrial respiration and ATP production. This also leads to an increase in cellular glutathione levels, thereby reducing ROS levels. The p53 protein has also been found to increase GLS2 expression, and this happens under both stress and nonstress conditions by increasing glutamate levels, increasing mitochondrial respiration and glutathione levels and decreasing ROS levels [99]. Although p53 promotes glycolysis and inhibits respiration in pancreatic β-cells and hepatocytes, it can also interestingly inhibit glycolysis by regulating the transcription of genes that affect glycolysis (RRAD, PFKFB3/4, TIGAR and the gene encoding the MCT1 transporter) [100]. One study showed that the loss of p53 in prostate cancer cells is responsible for the increased expression of HK2, which contributes to aerobic glycolysis [101]. Conversely, the loss of p53 results in the increased expression and activity of PGAM1 (Phosphoglycerate Mutase 1), thereby increasing the levels of glycolysis and biosynthesis required for tumour growth [102]. Interestingly, HK2 and PGAM1 are among the glycolytic enzymes silenced by p53 [99]. Furthermore, it appears that the expression of the glucose transporters GLUT1 and GLUT4 can be silenced by wild-type p53, while mutant forms of p53 induce in tumour cells an increase in the expression of these transporters and, consequently, an increase in glucose consumption associated with the Warburg effect [103].

There is an interaction between Akt and p53. The induction of p53 leads to a strong inhibition of Akt signalling through the activation of phosphatase and tensin homolog deleted on chromosome 10 (PTEN) transcription—it hydrolyses phosphatidylinositol (3,4,5)-trisphosphate (PIP3), which binds and activates Akt [104]. Parkin deficiency leads to the downregulation of the PTEN protein via S-nitrosylation and ubiquitination, leading to the activation of PI3K/Akt signalling in cells. p53 negatively regulates PI3K/Akt signalling through the induction of PTEN and parkin, which, in turn, inhibits glycolysis [105].

## 8. The Main Energy Source in a Cancer Cell

### 8.1. Glucose and Its Metabolism in Cancer

Glucose is the most common source of energy for cells and also a substrate for many biochemical processes. It can be produced by a gluconeogenesis process, occurring mainly in liver cells, or taken up by cells from the environment. The lipid membrane surrounding cells is completely impermeable to the glucose molecules, and the presence of transboundary transport proteins is essential. Glucose is transported to the cells by means of glucose transporters (GLUT) located in the cell membrane and then retained inside the cell by phosphorylation to glucose 6-phosphate. This process involves hexokinase and the ATP molecule [23,106,107]. Phosphorylated glucose is further phosphorylated to fructose-1,6-biphosphate using another ATP molecule. Then, the pyruvate produces acetyl-CoA, which reacts further in the tricarboxylic acid cycle. These transformations are accompanied by the production of two ATP molecules [2].

Due to the fact that cancer cells have an increased growth rate, some of them lack vital substances such as oxygen and glucose. The process of angiogenesis responds to the increased demand for these components, but the rate of this process is insufficient to meet the energy needs of a rapidly growing tumour [108]. In order to further grow and proliferate, tumour cells convert their metabolism from aerobic to anaerobic. Glycolysis is then the main source of ATP, and the pyruvate formed in this process is partially converted to lactic acid by lactate fermentation and excreted outside the cell. In response to an increased demand for glucose, cancer cells show, among other things, an increased expression of glucose transporters [109].

### 8.2. Glutamine in Cancer Metabolism

The other very important nutrient for a cancer cell is glutamine. This compound can, as a precursor of other amino acids, provide nitrogen atoms for the synthesis of nucleotide and deoxynucleotide bases. Curiously enough, according to research, 50% of nonessential amino acid (NEAA) necessary for the synthesis of proteins in cancer cells comes from glutamine [110,111].

Not only does glutamine contribute to the synthesis of nitrogenous bases and amino acids, but it is also a very important carbon donor for the synthesis of the acetyl-CoA molecule necessary for lipid synthesis. The studies showed that glutamine influences the TCA cycle through anaplerosis and supplements the necessary precursors for the synthesis of fatty acids [54,112].

Glutamine also influences redox homeostasis. Cancer cells are more exposed to ROSs, which are generated during electron transport in the mitochondrion. A certain amount of ROS is even necessary for the cell, but when the redox balance is disturbed, it can lead to macromolecular damage, which will inevitably lead to cell death. For this reason, cancer cells have developed a complex, intracellular system of antioxidant machinery that can dynamically provide reducing equivalents and remove ROS if necessary. One of the protection mechanisms of cancer cells against ROS is the increased presence of glutathione, which can directly eliminate hydrogen peroxide, or by using NADPH as a reducing agent needed to activate the antioxidant enzymes and to re-circulate glutathione [113,114,115].

Glutaminolysis plays a crucial role in the development of cancer, affecting the cell metabolism, growth, and signal pathways in the cell. Therefore, it is an attractive target in the strategy to fight cancer. The compounds that have shown effective effects are benzylserine and L-γ-glutamyl-p-nitroanilide (GPNA). These compounds are inhibitors of SLCA1A5 glutamine transporter. There is, however, a huge problem with the application of these agents—they are toxic to normal cells [116]. In response to this problem, small-molecule inhibitors were designed. These compounds include bis-2-(5-phenylacetamide-1,2,4-thiazole-2-yl)ethyl sulphide (BPTES), CB-839 and compound 968, which inhibit glutaminase (GLS) isoforms not found in normal cells. Unfortunately, BPTES, which inhibited tumour growth both in vivo and in vitro, is not suitable as a potential GLS inhibitor, due to its low solubility and poor bioavailability (Table 2) [117,118,119]. CB-839, which is a BPTES derivative, has a more selective effect as a GLS1 inhibitor. According to the research, it significantly influenced the development of tumours such as triple-negative breast cancer cells [120]. In turn, compound 968 showed anti-cancer effects against tumours that are highly resistant to chemotherapy, such as the brain, pancreas and breast cells [121]. Another interesting therapeutic proposal is the study of cells carrying KRAS mutations. Cells lacking glutamine arrest in the S and G2/M phases of the cell cycle and begin to show sensitivity to cytotoxic drugs. This represents a potential for further research in the fight against cancer cells acquiring resistance to applied therapies [122].

### 8.3. Glycolysis “Waste” Product

Lactate has been considered for many years as a waste product of glycolysis or glutaminolysis, but there are indications that this is an incorrect assumption. The amount of lactate in cancer cells can be up to 40 times higher, which is related to the aggressive nature of the cancer [123]. During the cancer development, lactic acid can act either as a fuel or a signalling molecule [124,125]. Furthermore, lactate is an important energy source for cancer cells when glucose is limited [126,127]. As described above, cancer cells can use glycolysis as one of their main energy sources while secreting large amounts of lactic acid. Interestingly, it has been observed that, although cancer cells may be near blood vessels with high oxygen levels, they prefer to use lactic acid, which reprograms cancer cells and promotes macrophage polarisation for a pro-inflammatory and pro-cancer phenotype. It can, therefore, be concluded that lactic acid is not a waste product of glycolysis, but may be an energy carrier between tumour cells and a protective molecule against harsh conditions [128]. Increasingly more attention is being paid to the cancer microenvironment, as it can play an important role in cancer development. Lactate is responsible for maintaining a low pH in the cancer microenvironment and, thus, contributes to: development of the disease, the invasion of cancer cells, angiogenesis and the weakness of the immune system [129,130].

The microenvironment of cancer consists of vascular endothelial cells, stromal cells, macrophages, lymphocytes and other cells of the immune system, of which fibroblasts can make up to 80% of the tumour mass [131]. The fibroblasts recruited by the tumour, referred to as cancer-related fibroblasts (CAF), can produce lactic acid, providing energy to the tumour cells and stimulating tumour proliferation [132].

The process of aerobic glycolysis leads to the transformation of pyruvate into lactic acid. This process is catalysed by lactic acid dehydrogenase—A [133]. The lactate accumulated in cancer cells is transported by MCT transporters. Special attention should be paid to MCT1 and MCT4 transporters, which may be used excessively to accelerate lactate secretion from the cytoplasm [134,135]. The consequence of this is a reduction in pH to 6.6 in the cancer microenvironment [136].

Lactic acid released from cancer cells activates VEGF. This factor stimulates cell division, proliferation and migration of endothelial cells. Lowering the oxygen level activates the hypoxia-inducing factor, which increases the expression of VEGF. Moreover, the process of angiogenesis is dependent not only on HIF-1a, but also on interleukin-8 which is formed by the stimulation of NFkB factor. This causes the formation of new blood vessels and the increased migration of endothelial cells [137].

As mentioned earlier, a large amount of lactate in a cell is associated with a decrease in immunity. Lactate formed in cancer cells may inhibit the activity of NK, dendritic and T cells [138,139]. What is particularly interesting is that research shows that low pH levels can affect the acquisition of resistance to existing cancer therapies. The main method of energy acquisition by a cancer cell is aerobic glycolysis. Due to a high glucose uptake and, thus, a higher lactic acid secretion by the cancer cell, the immune response is “suppressed” [140,141]. Therefore, lactic acid has become an attractive target in the fight against cancer.

### 8.4. Ketones and Fatty Acids in Cancer

Ketone bodies include acetoacetate, β-hydroxybutyrate and acetone. Ketones β-hydroxybutyrate, acetoacetate and acetone are very significant substrates for cancer cells [142]. These compounds are mainly observed in cells undergoing autophagy and in hepatocytes [143,144]. Although in adipocytes of cancer patients, there is increased lipolysis, the concentration of ketones in blood is not significantly increased. Ketone bodies may be secreted by CAFs, which suggests that ketones play an important role as substrates for cancer cells [144,145].

Interestingly, there are increasingly more reports suggesting that a ketogenic diet can support the fight against cancer. Some studies, where the influence of ketogenic diet on tumour development was studied, have shown that this diet can slow tumour growth, increase survival or sensitize certain types of tumours to existing therapies [146,147,148]. As many as 60% of the trials showed that ketogenic diet has an anticancer effect, while 10% of the research showed undesirable effects or increased cell proliferation [149]. The anticancer effect of ketogenic diet (KD) may be caused by the fact that, during diet, the level of blood glucose and the amount of the main product formed during aerobic glycolysis—lactic acid—in the cancer tissue decrease. The mechanism of action of ketogenic diet in the fight against cancer is very diverse, and it includes gene expression, as well as metabolism and the microenvironment of cancer-changed tissue [149,150].

Fatty acids are very important macromolecules because they are part of the membranes and they can be signal molecules responsible for cell growth, apoptosis or cell differentiation [151]. Fatty acids can be consumed and can also be synthesized de novo in the cell. Interestingly, fatty acids are an important source of energy for a cancer cell; they can provide 2.5 times more ATP than glucose oxidation [21]. Therefore, some types of cancer use fatty acids as an energy source and show an increased expression of enzymes necessary for their oxidation [151].

## 9. Crucial Transporters in Metabolism

### 9.1. Glucose Transporters

Glucose transporters belong to the superfamily of SLCs solute carrier transporters, among which 52 families of proteins regulating membrane transport, including inorganic ions, nucleotides, amino acids, neurotransmitters, sugars, purine bases and drug molecules, can be distinguished [152]. Proteins belonging to family of carriers SLC2A are responsible for transporting glucose. SLC2A is a family of 14 proteins identified so far, which have been grouped into three classes [153].

The SLC2A1 gene sequence in humans encodes the GLUT1 protein, which is located on the short arm of chromosome 1, in flight 1p35-31.3. Glucose transporter 1 is the best known and commonly occurring isoform of the GLUT family of proteins [154]. Transporter GLUT1 contains 12 hydrophobic α-helices connected to each other by short sections protruding above the cell membrane and forming a channel across it [155]. The N- and C-terminal regions of this protein are on the cytoplasmic side [156]. A characteristic feature of GLUT1 is the presence of two large loops between I and II, as well as the VI and VII transmembrane section. GLUT1 has an extracellular loop with a glycosylation site. In this loop at position 45, there is asparagine, which is subject to N-glycosylation. This modification affects protein stabilisation and, together with the O-glycosylation to which GLUT1 is also subject, enables full transport activity to be achieved [157]. Furthermore, the GLUT1 protein is characterized by the presence of positively charged RXGRR sequences, which are located in the second and eighth loop, allowing the protein to be properly embedded in the cell membrane. In the C-terminal region, you can find a DSQV motif that is responsible for binding the PDZ domain presented by GIPC proteins (Ga interacting protein, C-terminus); these proteins after binding to GLUT1 direct the transporter to the cell membrane and protect it from degradation in lysosomes. Located in loop 7 of the GLUT1 protein, the QLS sequence acts as a molecular filter, increasing its affinity for glucose [158,159].

The expression of GLUT protein in normal cells is characterized by strong tissue specificity. The overexpression of GLUT1 protein has been observed in the following cancers: liver, pancreas, breast, oesophagus, brain, kidneys, lungs, large intestine, ovaries and cervix [160]. In most cases, the overexpression of GLUT1 correlates with the stage of tumour progression and worse prognosis for the patient [160,161,162].

### 9.2. Monocarboxylate Transporters

MCT belongs to the SLC16A family of carriers, which includes 14 identified carrier proteins, of which only the first four are capable of catalysing proton-coupled monocarboxylates transport. These proteins are responsible for transport through membranes, molecules such as pyruvate, ketone bodies and lactic acid [163,164]. Monocarboxylate transporters play a key role in the metabolic adaptation of cancer. These transmembrane proteins are involved in the transport of lactate to sustain the hyperglycolytic phenotype and pH regulation in the cell to maintain the acid phenotype. Therefore, inhibition of monocarboxylate transporter activity is an attractive target in anticancer therapy [165].

MCT transporters play an essential part in cancer development. As mentioned earlier, the overexpression of MCT1 and MCT4 is associated with higher cancer aggressiveness and poorer prognosis in the patient [166,167]. An interesting issue is that MCT2 expression is associated with promising prognostic factors, namely the lack of metastases, low mitotic index and small tumour size, but surprisingly, MCT2 overexpression can be observed in lung cancer cells, the pancreas, prostate and colorectal [168].

## 10. Conclusions

Despite the rapid development of medical science, cancer remains one of the most pressing medical problems. Every year, more and more anti-cancer therapies appear in clinical practice, but still, no universal and effective method of cancer treatment has been developed. In recent years, compounds that affect not only cell structures, but above all, cellular metabolic processes are increasingly sought after.

It is well known that metabolism is an essential process in all cells. A characteristic feature of cancer cells is a high degree of proliferation, which is associated with a high demand for energy. Thanks to the discovery of Otto Warburg, attention has been paid to the metabolism of cancer cells over the last 100 years, which has allowed the discovery of new methods of treatment and new metabolic pathways. The discovery of previously unknown metabolic pathways allowed us to observe that cancer cells are able to modify their metabolism in such a way that they never run out of “fuel”. It turns out that the share of molecules considered as so-called “rubbish” in cancer metabolism is more significant than previously thought.

**Figure 3:**
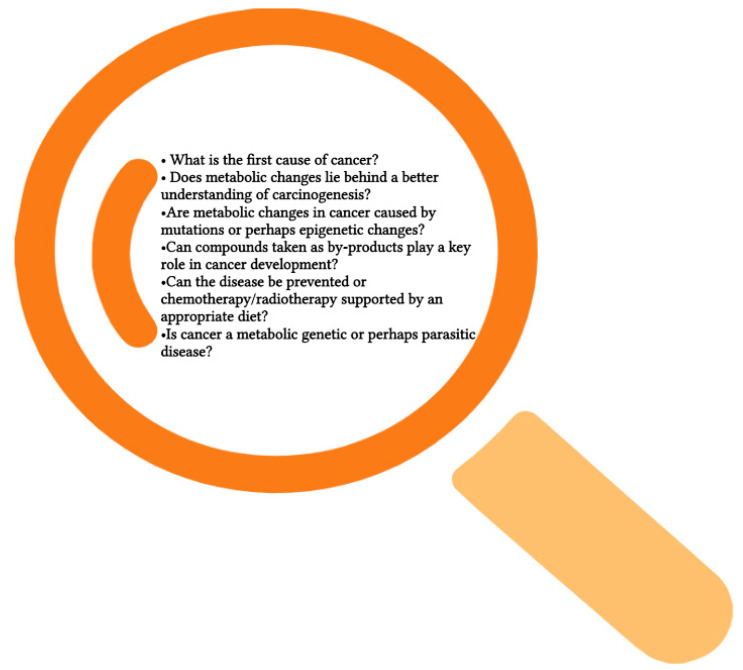
Graphical illustration of issues related to the heterogeneity of cancer metabolic phenotypes. Created with BioRender.com.

We are just beginning to understand the heterogeneity of metabolic phenotypes. It is likely that metabolic phenotypes may differ due to several factors: primary or metastatic tumour, tumour location, tumour microenvironment and mutation. A new emphasis on cancer metabolism may increase the development of metabolic inhibitors. While there has been a significant amount of progress in cancer metabolism research, there are still a number of questions on this subject, some of which are set out below (Figure 3).

There are more questions than answers at the moment, and we may be able to answer at least some of these questions in the coming years.

## Figures and Tables

**Table 1:** Overview of metabolic inhibitors in clinical trials.

Compound	Main Activity	Type of Cancer	References
CPI-613	inhibits pyruvate dehydrogenase (PDH) and a-ketogluterate dehydrogenase (KGDH)	Hematologic Malignancies	[77,78]
Olutasidenib (FT-2102)	Mutant isocitrate dehydrogenase 1 inhibitor	Acute Myeloid Leukemia	[79,80]
TVB-2640	Fatty Acid Synthase inhibitor	Non-small Cell Lung Cancer, Breast Cancer, Ovarian Cancer, Astrocytoma	[81,82]
Enasidenib (AG-221)	Mutant Isocitrate Dehydrogenase 2 Inhibitor	Acute Myeloid Leukemia	[83]
WZB117	GLUT1 inhibitor	Lung cancer, Breast cancer	[84,85]

**Table 2:** Classification of inhibitors of glutamine metabolism depending upon location of action.

Target	Drug	References
SLCA1A5	benzylserine	[116]
	GPNA	[116]
Glutaminase GLS1	BPTES	[117]
	CB-839	[118]
	compound 968	[119]

## Data Availability

Data sharing not applicable.

## Abbreviations

**Abbreviations**	**Meaning**
ACO	aconitase
ACSS2	acyl-coenzyme A synthetase short-chain family member 2
ADP	adenosine diphosphate
ANT	adenine nucleotide translocase
AST	aspartate aminotransferase
ATP	adenosine triphosphate
BPTES	bis-2-(5-phenylacetamide-1,2,4-thiazole-2-yl)ethyl sulfide
CAF	cancer-related fibroblasts
CD31	cluster of differentiation 31
c-Myc	c-Myc transcription factor
CO_2_	carbon dioxide
COX	cytochrome c oxidase
CSCs	cancer stem cells
DNA	deoxyribonucleic acid
ERK1/2	extracellular kinase 1/2
F-1,6-P	fructose 1,6-bisphosphate
F-2,6-BP	fructose-2,6-bisphosphate
F-6-P	fructose 6-phosphate
FAD	flavin adenine dinucleotide
FOXO3a	forkhead box protein O3
G3PH	glyceraldehyde 3-phosphate
G-6-P	glucose 6-phosphate
GLS	glutaminase
GLUD	glutamate dehydrogenase
GLUT	glucose transporters
GOT1/2	glutamate oxaloacetate transaminase 1/2
GPNA	L-γ-Glutamyl-p-nitroanilide
GSH	glutathione
HIF	hypoxia-inducible transcription factor
HO-1	heme oxygenase 1
IDH	isocytrine dehydrogenase
IL-8	interleukin 8
iNOS	induced nitric oxide synthase isoform
KD	ketogenic diet
KRAS	Kirsten rat sarcoma viral oncogene homolog
LDH	lactate dehydrogenase
LDHA	lactate dehydrogenase A
LDHB	lactate dehydrogenase B
L-Gln	L-glutamine
L-Glu	L-glutamic acid
MBP-1,	c-Myc binding protein 1
MCT	monocarboxylate transporter
mTOR	mammalian target of rapamycin
NAD	oxidised form of dinucleotide
NADPH	reduced form of NADP+
NEAA	non-essential amino acid
NFκB	nuclear factor kappa-light-chain-enhancer of activated B cells
NK	natural killer cells
OXPHOS	oxidative phosphorylation
OXPHOS	oxidative phosphorylation
p53	Tumour protein 53
PDH	pyruvate dehydrogenase
PDK1	pyruvate dehydrogenase kinase
PEP	phosphoenolpyruvate
PFK-2	phosphofructokinase 2
PFKFB	6-phosphofructo-2-kinase/fructose-2,6-biphosphatase 3
VEGF	vascular endothelial growth factor
